# Health Tract, No. 260

**Published:** 1866-09

**Authors:** 


### SUN-STROKE

Is an instantaneous inflammation of the brain, occasioned by the sun's rays communicating their heat to the structures with such intensity and rapidity as to cause dizziness, headache and nausea or vomiting; the patient then falls breathless, turns black in the face and dies, unless proper assistance is given on the spot; which is, to be taken to the shade. The neck should be instantly freed from all that binds it; pour warm water on the head, and dash it upon the body—the Arabs pour it in the ears, this may also be done. It is sometimes an hour or two before relief is obtained, which is ascertained by the patient becoming more conscious and more able to help himself. Let him drink as much water as he desires, if he can swallow it.

Sun-stroke is prevented by wearing a silk handkerchief in the crown of the hat, or green leaves, or a wet cloth of any kind; but during an attack warm water should be instantly poured on the head, or rags dipped in the water and renewed every minute. The reason is two-fold: the scalp is dry and hot, and the warm water not only removes the dryness, but carries off the extra heat with great rapidity, by evaporation. Sun-stroke is more common in the temperate than in the torrid zones. It is more frequent and fatal in New York and Quebec than in New Orleans and Havana. Day laborers are most liable to sun-stroke, especially in proportion as they use stimulating drinks. It is doubtful if any strictly temperate person ever becomes a victim to this instantaneous life-destroyer, but excessive exposure to the direct rays of a summer's sun, may occasion sun-stroke in any individual, in the proportion as he is of a sedentary occupation or of delicate health, Such persous, if compelled to be out of doors under a hot summer's sun, should wear a soft loose hat, with some light loose cloth in the crown; have the neck and throat bare and unconfined; should eat but little meat, and live mostly on coarse bread and butter and berries, ripe, raw and perfect, without sugar or milk, keep regular hours and have abundant sleep. Laborers should wash the whole scalp in cold water several times a day, and keep the surface of the body clean by rubbing it with a damp towel every night before going to bed. Let the friction be sufficiently vigorous to cause an extra redness of the skin. It is being between two fires that makes sun-stroke common in cities and uncommon on small islands or at sea, because the brick and stone pavements give back almost as great a heat as comes from the sun.

